# Enhanced Lithium Extraction from Brines: Prelithiation Effect of FePO_4_ with Size and Morphology Control

**DOI:** 10.1002/advs.202405176

**Published:** 2024-09-17

**Authors:** Xiaoyu Zhao, Shuo Yang, Xiuli Song, Yushuang Wang, Hui Zhang, Muhan Li, Yanfei Wang

**Affiliations:** ^1^ State Key Laboratory of Biobased Fiber Manufacturing Technology Tianjin University of Science and Technology Tianjin 300457 China; ^2^ Tianjin Key Laboratory of Brine Chemical Engineering and Resource Eco‐utilization College of Chemical Engineering and Materials Science Tianjin University of Science and Technology Tianjin 300457 China

**Keywords:** electrochemical lithium extraction, iron phosphate, low lithium sodium ratio, morphology, particle size, solid solution

## Abstract

Extracting lithium resources from seawater and brine can promote the development of the new energy materials industry. The electrochemical method is green and efficient. Iron phosphate (FePO_4_) crystal, with its 1D ion channel, holds significant potential as a primary lithium extraction electrode material. Li^+^ encounters a substantial concentration disadvantage in brines, and the co‐intercalation of Na^+^ diminishes Li^+^ selectivity. To address this issue, this work enhances the energy barrier for Na^+^ insertion through prelithiation strategies applied to the 1D channels of FePO_4_ crystal, thereby improving Li^+^ selectivity, and further investigating the prelithiation effect with particle size and morphology control. The results indicate that the Li_(4C‐40%)_FePO_4_// Activated carbon(AC) system enhances selectivity of lithium. The Li_(4C‐40%)_FePO_4_ with size diameter of 2500 nm demonstrates an energy consumption of 0.79 Wh mol^−1^ and a purity of 97.94% for lithium extraction at a unit lithium extraction of 5.93 mmol g^−1^ in simulated brine. Li_(4C‐40%)_FePO_4_‐nanoplates demonstrate the most optimal lithium extraction performance among the three morphologies due to their lamellar structure's short ion diffusion path in the [010] channel, favoring Li^+^ diffusion. The diffusion energy barriers of Li^+^ and Na^+^ are calculated using Density Functional Theory (DFT) before and after prelithiation, showing good agreement with experimental results.

## Graphical abstract

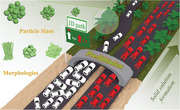

## 1 Introduction

Lithium, the lightest naturally occurring metal, is commonly known as the “energy metal of the 21st century” or likened to “white oil.” Given the swift progress in new energy vehicles, electronic devices, and energy storage technology, there has been a notable surge in interest regarding the utilization of lithium in the field of new energy materials.^[^ ^1^, ^2^, ^3^ ^]^ Numerous researchers and scholars have diligently pursued increased investment in the development of lithium batteries, resulting in remarkable achievements and significant progress.^[^ ^4^, ^5^, ^6^, ^7^, ^8^ ^]^ Despite the steady annual growth in lithium production, the market is still facing a scarcity issue that results in a significant imbalance. Hence, enhancing the lithium supply holds immense importance for the advancement of emerging industries.^[^ ^9^, ^10^ ^]^

The acquisition of lithium primarily involves two methods: solid lithium mining and the extraction of lithium from dissolved resources such as brine,^[^ ^11^ ^]^ underground brine,^[^ ^12^, ^13^ ^]^ and seawater.^[^ ^14^, ^15^ ^]^ The mining of ore resources leads to high energy consumption, high cost and high pollution issues making lithium extraction from dissolved resources is expected to be a major trend in the industry.^[^ ^16^, ^17^, ^18^, ^19^, ^20^ ^]^ Maximizing the utilization of dissolved lithium resources and reducing production costs are urgent needs for the development of new energy sources.^[^ ^9^, ^21^, ^22^, ^23^ ^]^

Several techniques have been successfully explored for extracting lithium from liquids, with the most widely used method being the evaporation process, which is known for its cost‐effectiveness and profitability. However, as it relies on solar evaporation, its efficiency and rate are significantly influenced by environmental factors, and the chemical treatment required after the initial evaporation results in the generation of a significant amount of waste.^[^ ^24^, ^25^ ^]^ Some new methods have been reported in recent years, including ion sieve adsorption, nanofiltration, solvent extraction, and electrochemical extraction. While ionic sieves exhibit high lithium adsorption capacity and selectivity, they are typically in powder form, leading to poor flowability and solution permeability, which ultimately results in low efficiency.^[^ ^26^, ^27^, ^28^, ^29^, ^30^ ^]^ Nanofiltration membranes utilize dielectric repulsion effect and spatial site resistance effect to hinder the passage of multivalent and large molecular weight compounds, but they are costly and complicated to operate.^[^ ^31^, ^32^, ^33^ ^]^ Due to the high extraction rate and low production cost, the solvent extraction method has obvious application advantages in brines of salt lakes with high Mg/Li ratios.^[^ ^34^, ^35^ ^]^ Nonetheless, the extraction process is susceptible to equipment piping corrosion, necessitating the use of high‐quality equipment materials.^[^ ^36^, ^37^ ^]^ Electrochemical lithium extraction is a new type of lithium extraction technology assisted by electric field, operating on principles similar to capacitive deionization.^[^ ^38^, ^39^, ^40^, ^41^, ^42^ ^]^ It has the advantages of high selectivity, low energy consumption, high efficiency and high energy utilization relative to the traditional lithium extraction methods, and does not require additional purification in operation, making it a lithium extraction technology with good industrial prospects.^[^ ^43^, ^44^, ^45^, ^46^, ^47^, ^48^, ^49^ ^]^

The FePO_4_ crystal, featuring a 1D ion channel, holds potential as a material for lithium storage and extraction.^[^ ^50^, ^51^, ^52^, ^53^ ^]^ Currently, there have been numerous researchers working on the application of lithium iron phosphate (LiFePO_4_) in the field of electrochemical lithium extraction, and significant results and progress have been achieved. Xiong et al. devised a method for extracting lithium from artificial feedstock water using LiFePO_4_/FePO_4_, achieving high separation coefficients.^[^ ^54^ ^]^ Wang et al. developed a unique electrochemical system for lithium extraction from brine using dual carbon source LiFePO_4_ (GC/IC/LFP) electrode materials, inspired by rocking chair lithium‐ion batteries.^[^ ^55^ ^]^ Du et al. extracted lithium from brine by reusing waste LiFePO_4_ powder, offering both a novel extraction method and an effective waste lithium recovery strategy.^[^ ^56^ ^]^ Zhu et al. investigated ion transport properties and rate‐determining steps in the electrode process kinetics of LiFePO_4_/Li_1‐x_FePO_4_ (0 < x < 1) using electrochemical impedance spectroscopy.^[^ ^57^ ^]^

The lithium sodium concentration ratio in seawater is generally low, and when it falls below a certain threshold, sodium tends to preferentially insert into the cathode material.^[^ ^58^, ^59^, ^60^ ^]^ All ions require a dehydrogenation process before embedding in FePO_4_, and Li^+^ has a significant dehydrogenation energy advantage over Mg^2+^ and Ca^2+^. Subsequently, the ions migrate to the voids within the lattice, with Li^+^ encountering a lower diffusion barrier compared to Na^+^, K^+^, and Mg^2+^. However, Li^+^ encounters a substantial concentration disadvantage in brines, and the co‐intercalation of Na^+^ diminishes Li^+^ selectivity.

**Figure 1:**
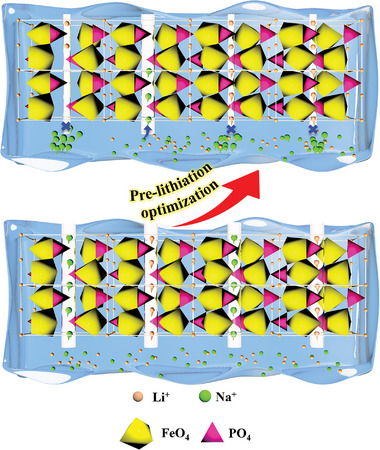
Illustration the enhancement of Li^+^ insertion advantage by prelithiation effect.

Preventing Na^+^ ingress can be achieved by increasing the energy barrier for Na^+^ insertion into the 1D channels of FePO_4_ crystals through the preinsertion of Li^+^, as illustrated in **Figure** **1**. This phenomenon of preinserting Li^+^ to enhance lithium selectivity was defined as the prelithiation effect.^[^ ^61^, ^62^ ^]^ The formation of solid solution by prelithiation of FePO_4_ is a multifaceted process, where particle size and morphology may influence the phase transition process during solid solution layer formation. Furthermore, solid solution layers formed under different prelithiation conditions may alter the extent of the prelithiation effect. Therefore, this study investigates the prelithiation effect of FePO_4_ with different particle sizes and morphologies.

## 2 Result and Discussion

### 2.1 Effect of Prelithiation

**Figure 2:**
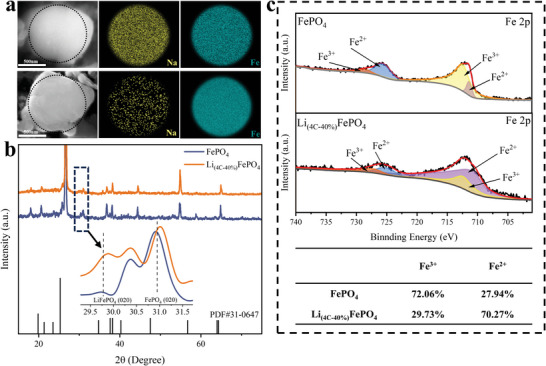
a) EDS mapping of FePO_4_ and Li_(4C‐40%)_FePO_4_ after co‐embedding Li^+^ and Na^+^, b) XRD patterns of FePO_4_ and Li_(4C‐40%)_FePO_4_, c) XPS spectra of Fe 2p in FePO_4_ and Li_(4C‐40%)_FePO_4_.

Yan et al. calculated the formation enthalpies of different structures with Li and Na co‐existence using DFT, demonstrating that Li and Na do not prefer to co‐exist in the same [010] channel during co‐interpolation.^[^ ^63^ ^]^ In order to further reveal the variation of elemental distribution in the materials, FePO_4_ and Li_(4C‐40%)_FePO_4_ electrodes were placed in a mixed solution (1 m NaCl, 10 mm LiCl) to embed Li^+^ and Na^+^ by applying a constant current, and the individual particles of both samples were subsequently characterized by SEM. As shown in **Figure** **2**a, the Fe element in both samples is uniformly distributed throughout the particles. Although EDS mapping cannot directly reveal lithium distribution, it can be inferred from the crystal structures of lithium iron phosphate and sodium iron phosphate, as well as the distributions of iron and sodium. EDS mapping of Li(4C‐40%)FePO4 shows uniform distribution of iron. The black areas outside the sodium distribution correspond to lithium. The higher proportion of black areas in the central region indicates that lithium is more concentrated in this area compared to sodium.^[^ ^64^, ^65^ ^]^ EDS mapping verified the calculation results experimentally, demonstrating the separation of the lithium and sodium phases and the high selectivity of the modified material for lithium.

The structural characteristics of FePO_4_ and Li_(4C‐40%)_FePO_4_ electrode materials were studied by XRD analysis. As shown in Figure 2b, both samples have the olivine structure with Pnma space group, which is consistent with the characteristic peaks of FePO_4_ standard card (JCPDS 31–0647). No impurity peaks were observed from the diffraction patterns, and all peaks were narrow and sharp, proving that the prepared samples were pure phases with good crystallinity. To better visualize subtle changes in the diffraction peaks of FePO_4_ and Li_(4C‐40%)_FePO_4_, the region between 29.5° and 31.5° was locally magnified. This magnification reveals significant alterations in the intensity bands between the characteristic (020) peaks of FePO_4_ and LiFePO_4_. Specifically, the lattice (020) peak position of LiFePO_4_ shifts to the right by 0.14°, accompanied by an 82% increase in peak intensity. These changes indicate a sustained structural transformation during the formation of the prelithiated material. The intensity band observed between the two final phases corresponds to the intermediate solid solution phase.^[^ ^63^ ^]^

The XPS full spectrum of Figure S4 (Supporting Information) shows that the positions of the characteristic peaks of FePO_4_ and Li_(4C‐40%)_FePO_4_ are consistent, which proves the existence of Fe, P, O, and C in both materials. And the elemental valence changes before and after prelithiation were confirmed by XPS as shown in Figure 2c. Due to spin‐orbit splitting, the spectrum of Fe 2p is split into two oxidation states, with significant changes in the Fe valence share before and after prelithiation.^[^ ^66^ ^]^ In FePO_4_, the binding energies of Fe^3+^ are 728.48 and 712.48 eV, respectively, with a total share of 72.06%, and the binding energies of Fe^2+^ are 727.78 and 711.18 eV, respectively, with a total share of 27.94%. In Li_(4C‐40%)_FePO_4_, the binding energies of Fe^3+^ were 727.58 and 712.58 eV, respectively, with a total share of 29.73%, and the binding energies of Fe^2+^ were 725.28 and 711.28 eV, respectively, with a total share of 70.27%. This change in data matches the expected effect of the experiment and further validates the successful implementation of the prelithiation process.

**Figure 3:**
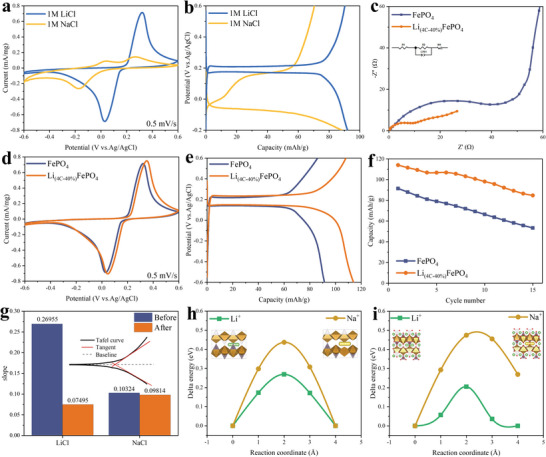
a) CV tests for the FePO_4_ electrodes in 50 mL 1 m LiCl/NaCl aqueous solution at a 0.5 mV s^−1^ scan rate (paired with Ag/AgCl reference). b) Comparison of electrochemical cycling of the FePO_4_ electrodes in 50 mL 1 m LiCl/NaCl aqueous solutions under 1C, using Ag/AgCl as the reference electrodes. c) EIS curves of FePO_4_ and Li_(4C‐40%)_FePO_4_ electrodes. d) CV tests for the FePO_4_ and Li_(4C‐40%)_FePO_4_ electrodes in 50 mL 1 m LiCl aqueous solution at a 0.5 mV s^−1^ scan rate (paired with Ag/AgCl reference). e) Comparison of electrochemical cycling of the FePO_4_ and Li_(4C‐40%)_FePO_4_ electrodes in 50 mL 1 m LiCl aqueous solutions under 1C, using Ag/AgCl as the reference electrodes. f) Discharge capacity for 15 cycles of FePO_4_ and Li_(4C‐40%)_FePO_4_. g) Test results of Tafel of FePO_4_ and Li_(4C‐40%)_FePO_4_. Simulated diffusion path and diffusion energy barrier of Li^+^ and Na^+^ on the surface of the h) FePO_4_ and i) Li_(4C‐40%)_FePO_4_. [Correction added on 25 October 2024, after first online publication: Figure 3 is replaced with updated version.]

**Figure** **3**a shows the results of cyclic voltammetry tests of FePO_4_ electrodes in 1 m LiCl and 1 m NaCl solutions at a scan rate of 0.5 mV s^−1^, respectively. The CV curves of both show obvious oxidation and reduction peaks, proving that both lithium and sodium ions can compete to embed in the FePO_4_ host. Figure 3b shows the results of cyclic charge‐discharge tests of FePO_4_ electrode in 1 m LiCl and 1 m NaCl solutions at 1 C, respectively. The charge‐discharge curves of both have a clear potential plateau in the voltage range of −0.6–0.6 V, which is consistent with the peak of the CV curve.^[^ ^67^, ^68^ ^]^

The EIS curves and equivalent circuit fitting for FePO_4_ and Li_(4C‐40%)_FePO_4_ are illustrated in Figure 3c. Comparing the electrolyte resistance (R1) and charge transfer resistance (R2), it's evident that Li_(4C‐40%)_FePO_4_ exhibits lower values (R1: 11.33 Ω, R2: 3.94 Ω) than those of the original FePO_4_ (R1: 61.34 Ω, R2: 35.10 Ω). This indicates that the prelithiation can improve electron conductivity, ion transport rate, reducing hindrance to the diffusion and surface transfer of Li^+^.

Figure 3d showcases the results of CV tests conducted on Li_(4C‐40%)_FePO_4_ and FePO_4_ electrodes immersed in a 1 m LiCl solution at a scan rate of 0.5 mV s^−1^. In all CV curves, a pair of distinct redox peaks can be observed, corresponding to the intercalation and deintercalation processes of Li^+^ ions, respectively. Notably, Li_(4C‐40%)_FePO_4_ exhibits broader peak widths and higher peak current values compared to FePO_4_. This suggests that the early insertion of Li^+^ facilitates the formation of additional Li^+^ diffusion channels, promoting rapid diffusion within the material and enhancing the electrochemical reaction rate. Consequently, the ion intercalation capacity is improved, leading to increased capacity.

Figure 3e shows the results of cyclic charge‐discharge tests of Li_(4C‐40%)_FePO_4_ and FePO_4_ electrodes in 1 m LiCl solution at 1C. FePO₄ and Li_(4C‐40%)_FePO_4_ were pretreated in 50 mL of 1 m LiCl, charged at 1 C to 0.6 V for lithium removal before the constant current charge‐discharge test. The charging and discharging curves of both have a clear potential plateau in the voltage range of −0.6–0.6 V, which is consistent with the peak of the CV curve and the first‐turn discharge specific capacity of Li_(4C‐40%)_FePO_4_ is larger than that of pristine FePO_4_. The variation of the discharge specific capacity of FePO_4_ and Li_(4C‐40%)_FePO_4_ for 15 cycles at 1 C is shown in Figure 3f. Electrode materials may exhibit fluctuating discharge specific capacity during the initial activation cycles. The discharge specific capacity of FePO_4_ was 91.5 mAh g^−1^ in the first cycle and 53.4 mAh g^−1^ after 15 cycles, maintaining 58.18% of the initial capacity, while the discharge specific capacity of Li_(4C‐40%)_FePO_4_ was 114.1 mAh g^−1^ in the first cycle and 84.8 mAh g^−1^ after 15 cycles, maintaining 74.32% of the initial capacity. The capacity and cycling performance of Li_(4C‐40%)_FePO_4_ electrode is significantly better than that of FePO_4_, which is due to the fact that during the prelithiation process, Li^+^ enter the lattice of the material and form stable lithium storage sites. These sites are capable of reversibly embedding and de‐embedding Li^+^ during charging and discharging, which reduces disordered Li^+^ buildup and diffusion, and thus improves the structural stability and cycling stability of the electrodes.

Figure S5 (Supporting Information) displays the Tafel curve test outcomes of Li_(4C‐40%)_FePO_4_ and FePO_4_ in 1 m LiCl solution and 1 m NaCl solution, respectively. A comparison of the fitting results depicted in Figure 3g reveals a significant increase in the diffusion rate of Li^+^ relative to sodium ions following the prelithiation process. The diffusion energy barriers of Li^+^ and Na^+^, both before and after prelithiation, were calculated utilizing DFT. Ions diffusing between different sites in a material need to overcome certain energy barriers to reach a stable state. Three intermediate states were set between neighboring insertion sites, and the dynamic diffusion path was shown in Figure S6 (Supporting Information). Figure 3h shows the diffusion energy barrier distribution of Li^+^ and Na^+^ in FePO_4_, the energy required for Li^+^ diffusion is 0.270 eV, whereas it is 0.437 eV for Na^+^. Figure 3i shows the diffusion energy barrier distribution of Li^+^ and Na^+^ in Li_(4C‐40%)_FePO_4_, the energy required for Li^+^ diffusion is 0.205 eV, and it is 0.485 eV for Na^+^. These results indicate that the prelithiation process decreased the diffusion barrier of Li^+^ by 0.065 eV and increased the diffusion barrier of Na^+^ by 0.048 eV, which is consistent with the experimental results.^[^ ^69^, ^70^ ^]^

### 2.2 Prelithiation Effect with Particle Size

**Figure 4:**
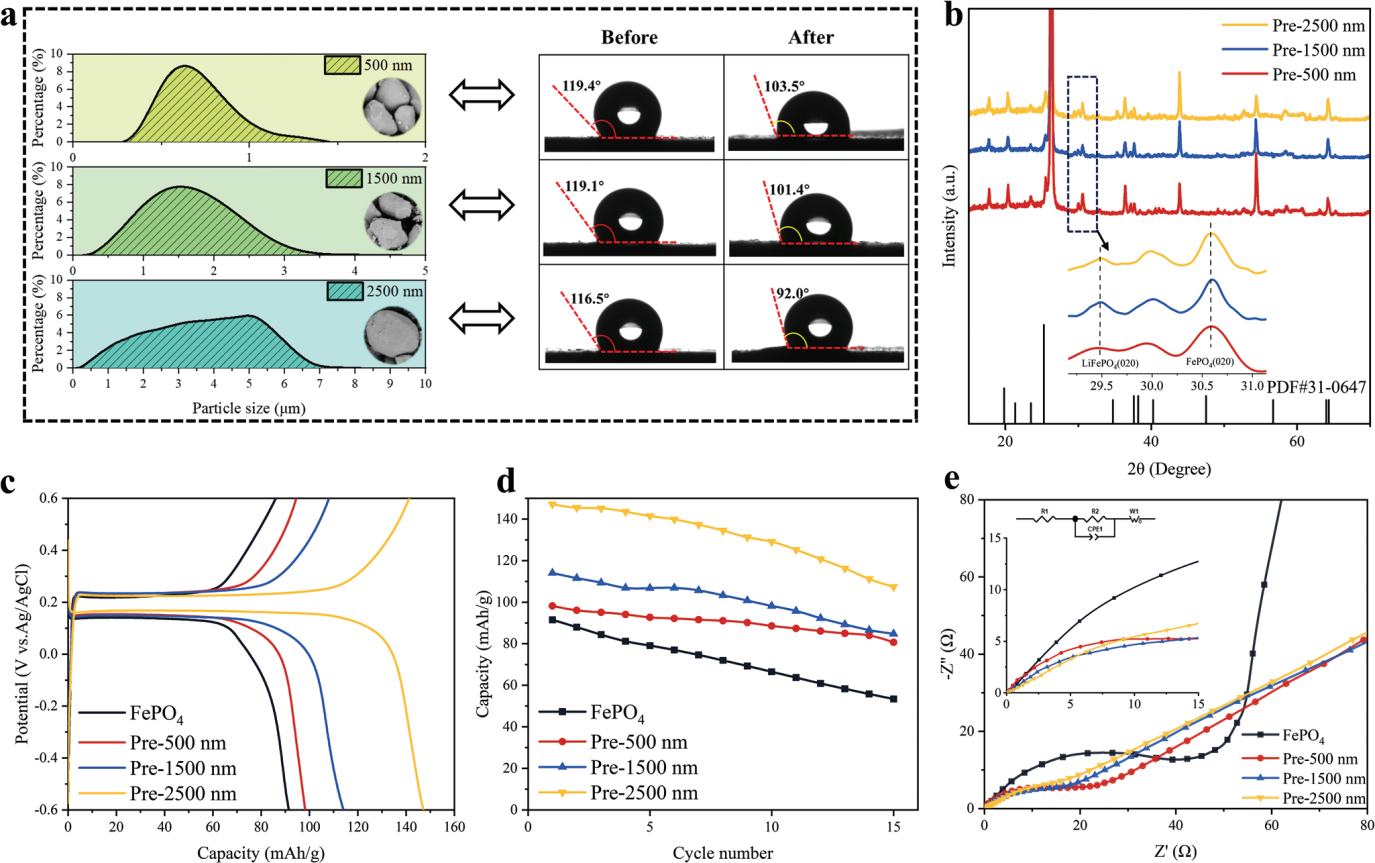
a) Particle size distribution and contact Angle of FePO_4_ (500, 1500, and 2500 nm). b) XRD patterns of Li_(4C‐40%)_FePO_4_ (500, 1500, and 2500 nm). c) Comparison of electrochemical cycling of the FePO_4_ and Li_(4C‐40%)_FePO_4_ (500, 1500, and 2500 nm) electrodes in 50 mL 1 m LiCl aqueous solutions under 1C, using Ag/AgCl as the reference electrodes. d) Discharge capacity for 15 cycles of FePO_4_ and Li_(4C‐40%)_FePO_4_ (500, 1500, and 2500 nm). e) EIS curves of FePO_4_ and Li_(4C‐40%)_FePO_4_ (500, 1500, and 2500 nm) electrodes.

Three different particle sizes (500, 1500, and 2500 nm) of FePO_4_ particles were modulated by controlling the experimental conditions, and their particle size distributions and contact angles are shown in **Figure** **4**a.^[^ ^71^ ^]^ Due to the existence of PVDF cladding layer, the electrode plate was hydrophobic (contact angle ≥116.5°), which makes the wettability of the electrode material poor. Specifically, the contact angle of FePO_4_‐500 is 119.4°, which is reduced to 103.5° for its corresponding Li_(4C‐40%)_FePO_4_‐500 after the prelithiation treatment; similarly, the contact angle of FePO_4_‐1500 is reduced from 119.1° to 101.4° for Li Li_(4C‐40%)_FePO_4_‐1500; and the contact angle of FePO_4_‐2500 decreased from 116.5° to 92.0° for Li_(4C‐40%)_FePO_4_‐2500. The contact angles of Li_(4C‐40%)_FePO_4_ are smaller than those of FePO_4_, indicating the hydrophilicity of the electrode was significantly enhanced by the modification via the prelithiation. Larger particles expose more surface active sites, which enhances the adsorption of solvent molecules thus increases the hydrophilicity. Additionally, during prelithiation, the crystal structure of lithium iron phosphate particles adjusts, revealing more active sites that interact effectively with water molecules, further boosting hydrophilicity. Enhanced hydrophilicity implies a larger contact area between the electrode material and the electrolyte, thereby improving the ionic conductivity, which is directly related to the capacity and cycling performance of the system. Figure 4b shows the XRD results of Li_(4C‐40%)_FePO_4_ with different particle sizes. All materials exhibit the olivine structure of the Pnma space group, consistent with the characteristic peaks of the FePO_4_ standard card (JCPDS 31–0647). The diffraction patterns did not reveal any other impurities, and the peaks are narrow and sharp, indicating that the prepared samples are pure phases with good crystallinity. To illustrate the detailed variations in the FePO_4_ and Li_(4C‐40%)_FePO_4_ diffraction peaks, local magnification between 29.5° and 31.5° was performed. The variation in the intensity bands between the characteristic (020) peaks of FePO_4_ and LiFePO_4_ suggests the successful formation of the prelithiated solid solution.

The variation of discharge specific capacity of FePO_4_ and Li_(4C‐40%)_FePO_4_ for 15 cycles at 1C is shown in Figure 4d. Among them, the discharge specific capacity of Li_(4C‐40%)_FePO_4_‐500 is 98.2 mAh g^−1^ for the first cycle, Li_(4C‐40%)_FePO_4_‐1500 is 114.1 mAh g^−1^ for the first cycle, and Li_(4C‐40%)_FePO_4_‐2500 is 147.2 mAh g^−1^ for the first cycle. It is clearly seen from the figure that the prelithiated electrode material Li_(4C‐40%)_FePO_4_ exhibits a higher discharge specific capacity than FePO_4_ at all particle sizes, and it increases with the increase of particle size. Larger particle sizes of FePO_4_ particles usually have better electron conduction properties, which facilitates the formation of a continuous electron transport path in the electrode material and makes the prelithiation process more adequate. This helps to improve the electrical conductivity of the electrode material and increase the charge transfer efficiency, thus improving the electrochemical performance.

Figure 4e illustrates the EIS curves of FePO_4_ and Li_(4C‐40%)_FePO_4_ with varying particle sizes. After equivalent circuit fitting, it was observed that the electrolyte resistance R1 and charge transfer resistance R2 of Li_(4C‐40%)_FePO_4_ are smaller than those of pristine FePO_4_. Specifically, for Li_(4C‐40%)_FePO_4_‐500, the electrolyte resistance R1 is 37.85 Ω, and the charge transfer resistance R2 is 18.93 Ω; for Li_(4C‐40%)_FePO_4_‐1500, R1 is 29.38 Ω, and R2 is 10.31 Ω; for Li_(4C‐40%)_FePO_4_‐2500, R1 is 12.64 Ω, and R2 is 8.51 Ω. These findings suggest that the prelithiation process of materials with different particle sizes reduces the energy barrier for Li^+^ de‐embedding within the materials, with resistance decreasing as particle size increases. This can be attributed to the larger channels and gaps in the electrode sheets coated with larger particle sizes, allowing ions to diffuse more easily within the particles. Furthermore, preinserted lithium ions can more easily diffuse and embed within the particles, resulting in a more uniform distribution of ions within the particles. Additionally, more lithium ions can bind with the active sites inside the particles, forming a stable prelithiated solid solution layer. Therefore, after prelithiation treatment, electrode sheets made from larger particle sizes exhibit a more significant prelithiation effect.

The CV results of FePO_4_ and Li_(4C‐40%)_FePO_4_ with different particle sizes in 1 m LiCl solution are shown in Figure S7 (Supporting Information), respectively. A set of redox peaks are present for all materials, representing the de‐embedding of Li^+^ from the material in one step. With the increase of sweep speed, both higher peak currents and peak potentials appeared in the CV curves, and the oxidation and reduction peak potential difference increased. Their diffusion coefficients during oxidation and reduction processes are shown in **Table** **1**. The diffusion coefficients of Li_(4C‐40%)_FePO_4_ are higher than those of FePO_4_, indicating that the prelithiation process effectively improved the Li^+^ transport rate.

**Table 1:** Diffusion coefficients in FePO_4_ and Li_(4C‐40%)_FePO_4_ (500, 1500, and 2500 nm) electrodes in 1 m LiCl electrolyte.

Material	O_x1_[cm^2^ s^−1^]	*R*_ed1_[cm^2^ s^−1^]
FePO_4_	6.08 × 10^−12^	5.66 × 10^−12^
Li_(4C‐40%)_FePO_4_‐500	1.13 × 10^−11^	1.03 × 10^−11^
Li_(4C‐40%)_FePO_4_‐1500	1.98 × 10^−11^	1.96 × 10^−11^
Li_(4C‐40%)_FePO_4_‐2500	3.03 × 10^−11^	2.89 × 10^−11^

The rate capability of FePO_4_ and Li_(4C‐40%)_FePO_4_ at charging and discharging rates ranging from 0.5 C to 4 C is illustrated in Figure S8 (Supporting Information). Li_(4C‐40%)_FePO_4_ consistently exhibits higher discharge specific capacity compared to FePO_4_ across various charging rates. The benefits of the prelithiated structure become more evident as the charge/discharge rate increases. Initially, FePO_4_ and Li_(4C‐40%)_FePO_4_ show capacities of 124.5 and 150.8 mAh g^−1^ (0.5 C), respectively. Upon returning to the initial rate after testing, FePO_4_ retains only 58.2 mAh g^−1^, while Li_(4C‐40%)_FePO_4_ maintains 106.8 mAh g^−1^. The accelerated Li^+^ diffusion rate within the olivine phase contributes significantly to the enhanced performance. Furthermore, the formation of the prelithiated structure effectively stabilizes the material's surface structure and mitigates irreversible capacity decay.

**Figure 5:**
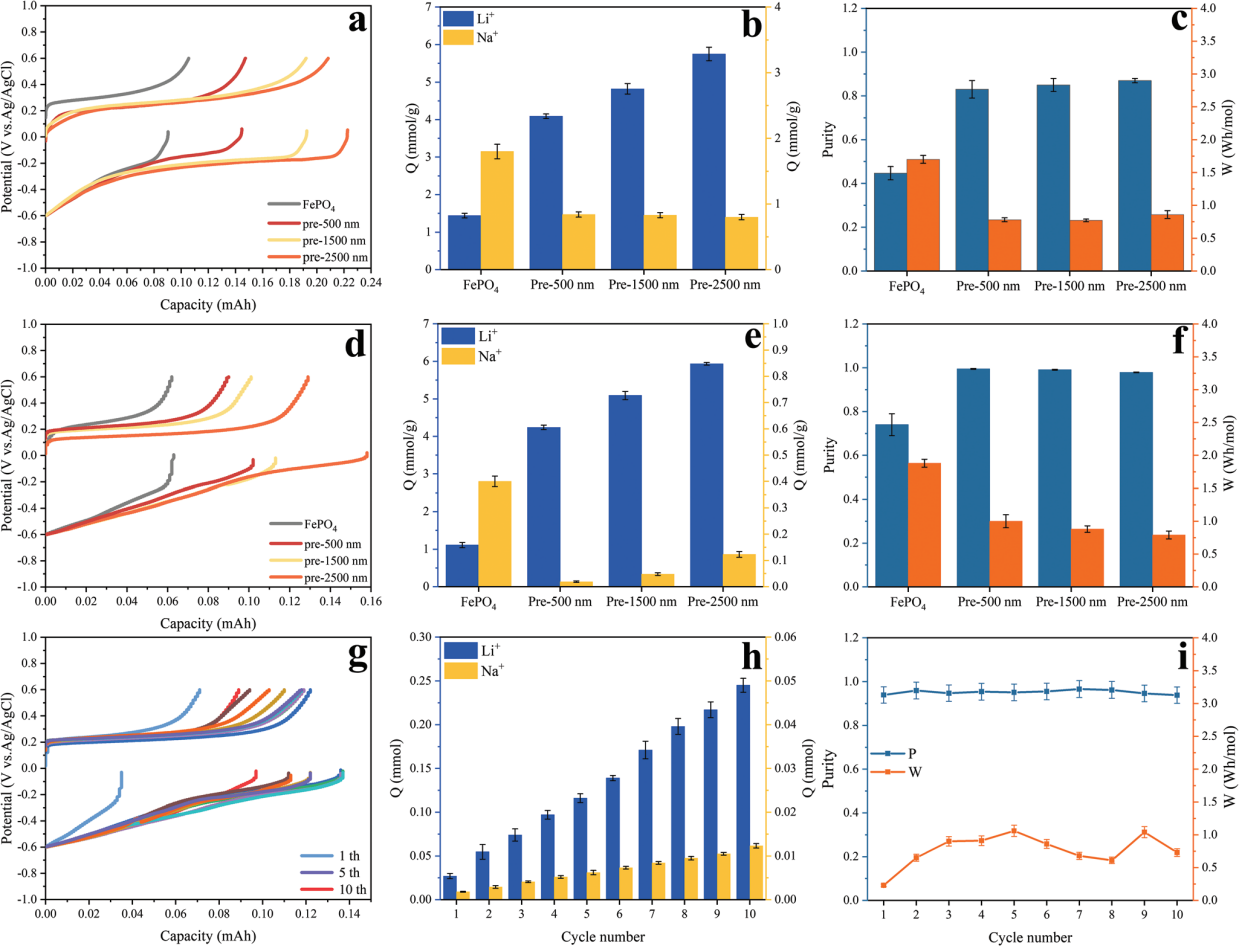
FePO_4_ and Li_(4C‐40%)_FePO_4_ (500, 1500, and 2500 nm) in 1 m NaCl and 10 mm LiCl electrolyte to a) Potential versus charge curves, b) Li^+^ and Na^+^ extraction amount in receiving solution and c) corresponding Li^+^ extraction purity and molar consumption. FePO_4_ and Li_(4C‐40%)_FePO_4_(500, 1500, and 2500 nm) in Uyuni brine to d) Potential versus charge curves, e) Li^+^ and Na^+^ extraction amount in receiving solution and f) corresponding Li^+^ extraction purity and molar consumption Li_(4C‐40%)_FePO_4_‐1500 in Uyuni brine for 10 cycles of lithium extraction cycle of g) Potential versus charge curves, h) gradual accumulation of Li^+^ and Na^+^ at the receiving electrolyte solution and i) corresponding Li^+^ extraction purity and molar consumption.

FePO_4_ and Li_(4C‐40%)_FePO_4_ were pretreated for lithium removal by charging at 1 C to 0.6 V in 50 mL of 1 m LiCl prior to the lithium extraction test. **Figure** **5**a shows the potential and capacity curves of the FePO_4_//AC and Li_(4C‐40%)_FePO_4_//AC systems with different particle sizes in a mixed solution with a lithium to sodium ratio of 0.01(1 m NaCl and 10 mm LiCl). Li_(4C‐40%)_FePO_4_ exhibits superior lithium extraction performance in low lithium to sodium ratio solutions, and the unit lithium extraction increased with the increase of particle size. The lithium extraction for one cycle of FePO_4_ was 1.44 mmol/g^−1^, while Li_(4C‐40%)_FePO_4_‐500, Li_(4C‐40%)_FePO_4_‐1500, and Li_(4C‐40%)_FePO_4_‐2500 reached 4.09, 4.82, and 5.75 mmol g^−1^, respectively (Figure 5b). The larger particle size of Li_(4C‐40%)_FePO_4_ has better electron conduction properties, which facilitates the formation of a continuous electron transport path in the electrode material, thus lowering the lithium ion embedding energy barrier. The energy consumption per unit of Li_(4C‐40%)_FePO_4_ with different particle sizes was less than that of FePO_4_, and 1.71 Wh mol^−1^ for one cycle of FePO_4_, 0.78 Wh mol^−1^ for one cycle of Li_(4C‐40%)_FePO_4_‐500, 0.77 Wh mol^−1^ for one cycle of Li_(4C‐40%)_FePO_4_‐1500, and 0.86 Wh mol^−1^ for one cycle of Li_(4C‐40%)_FePO_4_‐2500. In addition, the purity of lithium extraction of Li_(4C‐40%)_FePO_4_ was higher than that of FePO_4_ (44.37%) for different particle sizes (82.96%, 85.34%, and 87.74%) (Figure 5c).

Figure 5d shows potential and capacity curves of the FePO_4_//AC and Li_(4C‐40%)_FePO_4_//AC systems with different particle sizes in simulated brine at Uyuni(23.48 mm Li^+^, 120.83 mm Mg^2+^, 256.4 mm Na^+^, 47.83 mm K^+^, and 0.57 mm Ca^2+^). The lithium extraction per unit of Li_(4C‐40%)_FePO_4_ is greater than FePO_4_ for different particle sizes. Specifically, the lithium extraction of one cycle of FePO_4_ was only 1.11 mmol g^−1^, while Li_(4C‐40%)_FePO_4_‐500, Li_(4C‐40%)_FePO_4_‐1500, and Li_(4C‐40%)_FePO_4_‐2500 had lithium extraction as high as 4.24, 5.09, and 5.93 mmol g^−1^, showing an increasing trend with increasing particle size (Figure 5e). The energy consumption for lithium extraction in one cycle of FePO_4_ was 1.88 Wh mol^−1^, while that of Li_(4C‐40%)_FePO_4_‐500, Li_(4C‐40%)_FePO_4_‐1500, and Li_(4C‐40%)_FePO_4_‐2500 was reduced to 1.01, 0.88, and 0.79 Wh mol^−1^, indicating that the prelithiation treatment not only enhanced the lithium extraction efficiency but also reduced the energy consumption. This can be attributed to the prelithiation treatment reducing the competitive insertion of sodium ions, enhancing the selectivity and diffusion rate of lithium, thereby expanding the electrochemical kinetics advantage of lithium insertion process. The purity of lithium extraction of Li_(4C‐40%)_FePO_4_ with different particle sizes (99.54%, 99.06%, and 97.94%) were greater than FePO_4_ (73.75%) (Figure 5f).

The results were also systematically compared with the lithium extraction performance of other published electrode systems, as shown in **Table** **2**. The extraction capacity of Li_(4C‐40%)_FePO_4_//AC was better than most of the published work, reaching up to 5.93 mmol g^−1^. As for energy consumption, Li_(4C‐40%)_FePO_4_//AC showed superior values which is more efficient than reported systems. In terms of Li^+^ selectivity, the separation coefficient of the Li_(4C‐40%)_FePO_4_//AC system reached 2389, which is higher than other systems. So, Li_(4C‐40%)_FePO_4_//AC showed a competitive performance in electrochemical Li^+^ recovery from brines after comprehensive comparison.

**Table 2:** Comparision of Li_(4C‐40%)_FePO_4_//AC among the other reported electrode materials on electrochemical performance.

Electrode	Raw Solution	Energy Consumption [Wh mol^−1^]	Capacity [mmol g^−1^]	Selectivity	Refs.
LiFePO_4_/FePO_4_(Carbon fiber cloth)	Yiliping raw brine solution	n.a.	3.57	λ_Li‐Mg(calculated)_ = 97	[72]
LiFePO_4_/Pt	0.2 mol L^−1^ Li^+^ + 4.5 mol L^−1^ Na^+^	5.38	2.09	λ_Li‐Na_ = 210.5	[56]
LiFePO_4_/FePO_4_(Porous foam nickel)	West Taijnar salt lake	n.a.	5.56	λ_Li‐Mg(calculated)_ = 133	[73]
LiFePO_4_/Porous carbon	50 ppm Li^+^ solution	n.a.	1.59	n.a.	[74]
LiFePO_4_/FePO_4_(Control the electrolytic voltage)	0.2 g L^−1^ Li^+^ and 0.5 mol L^−1^ Na^+^ mixed chloride solution	n.a.	5.51	n.a.	[75]
LiFePO_4_/FePO_4_(Continuous nitrogen‐flushing)	5 mm LiCl + 50 mm NaCl solution	3.03 ± 0.5	3	n.a.	[76]
FePO_4_//AC	Uyuni salt lake	1.88	1.11	λ_Li‐Na_ = 31	This work
Li_(4C‐40%)_FePO_4_‐500//AC		1.01	4.24	λ_Li‐Na_ = 2389	
Li_(4C‐40%)_FePO_4_‐1500//AC		0.88	5.09	λ_Li‐Na_ = 1148	
Li_(4C‐40%)_FePO_4_‐2500//AC		0.79	5.93	λ_Li‐Na_ = 519	
Li_(4C‐40%)_FePO_4_‐nanoparticles//AC		0.96	2.40	λ_Li‐Na_ = 361	
Li_(4C‐40%)_FePO_4_‐nanorods//AC		0.80	3.76	λ_Li‐Na_ = 775	
Li_(4C‐40%)_FePO_4_‐nanoplates//AC		1.13	5.06	λ_Li‐Na_ = 945	

Li_(4C‐40%)_FePO_4_‐1500//AC systems was subjected to 10 cycles of lithium extraction experiments in simulated brine, and the potential and capacity curves are shown in Figure 5g. It has stable lithium extraction ability in simulated brine, and the content of Li^+^ in the receiving solution increases with the number of cycles, which is much higher than the growth rate of the content of sodium ions (Figure 5h). The purity of Li^+^ in the receiving solution was 93.86% after the first cycle and 93.85% after 10 cycles (Figure 5i). It indicates that the Li_(4C‐40%)_FePO_4_ system can effectively and selectively separate Li^+^ from other cations with long term stability.

### 2.3 Prelithiation Effect with Morphology

**Figure 6:**
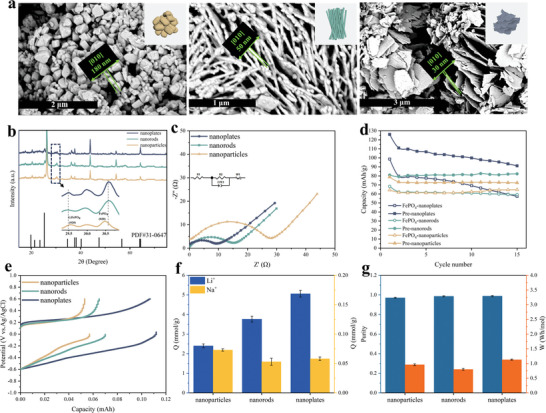
a) SEM images of Li_(4C‐40%)_FePO_4_(nanoparticles, nanorods, nanoplates). b) XRD patterns of Li_(4C‐40%)_ FePO_4_ (nanoparticles, nanorods, and nanoplates). c) EIS curves of Li_(4C‐40%)_FePO_4_(nanoparticles, nanorods, nanoplates) electrodes. d) Discharge capacity for 15 cycles of FePO_4_ and Li_(4C‐40%)_FePO_4_(nanoparticles, nanorods, and nanoplates). Li_(4C‐40%)_FePO_4_(nanoparticles, nanorods, and nanoplates) in Uyuni brine to e) Potential versus charge curves, f) Li^+^ and Na^+^ extraction amount in receiving solution, and g) corresponding Li^+^ extraction purity and molar consumption.

LiFePO_4_ particles with three different morphologies (nanoparticles, nanorods, and nanoplates) were synthesized via a hydrothermal method, as depicted in **Figure** **6**a. The key determinant influencing the electrochemical properties of the three materials with distinct morphologies is the length of the ion transport path along the [010] direction. EDS mapping (Figure S9, Supporting Information) confirmed the homogeneous distribution of Fe, P, and O elements in LiFePO_4_. Subsequently, Li_(4C‐40%)_FePO_4_ with various morphologies were prepared through same method used previously. Mapping analysis (Figure S10, Supporting Information) revealed uniform distribution of all elements, with sodium exhibiting a relatively sparse distribution compared to other elements. Figure 6b shows the XRD results of Li_(4C‐40%)_FePO_4_ with different morphologies. All materials have the olivine structure of the Pnma space group, which is in general agreement with the characteristic peaks of the FePO_4_ standard card (JCPDS 31–0647). The diffraction pattern did not reveal any other impurities and the peaks were narrow and sharp, indicating that the prepared samples were pure phases and well crystallized. In order to show the detailed variations of the FePO_4_ and Li_(4C‐40%)_FePO_4_ diffraction peaks, the local zoom between 29.5° and 31.5° was performed, and the variation of the intensity bands between the characteristic (020) peaks of FePO_4_ and LiFePO_4_ indicated the successful formation of the prelithiated solid solution layer.

The CV curves of different morphologies of Li_(4C‐40%)_FePO_4_ in 1 m LiCl solution at different scan rates are shown in Figure S11 (Supporting Information). The peak potential difference also increases with increasing scan rate due to concentration polarization and electrochemical polarization. Their diffusion coefficients during oxidation and reduction processes are shown in **Table** **3**. Li_(4C‐40%)_FePO_4_‐nanoplates demonstrate the most optimal performance among the three morphologies due to their lamellar structure's short ion diffusion path in the [010] channel, favoring Li^+^ diffusion.

**Table 3:** Diffusion coefficients in Li_(4C‐40%)_FePO_4_(nanoparticles, nanorods, nanoplates) electrodes in 1 m LiCl electrolyte.

Material	O_x1_ [cm^2^ s^−1^]	R_ed1_ [cm^2^ s^−1^]
Li_(4C‐40%)_FePO_4_‐nanoparticles	8.43 × 10^−12^	6.78 × 10^−12^
Li_(4C‐40%)_FePO_4_‐nanorods	2.08 × 10^−11^	1.38 × 10^−11^
Li_(4C‐40%)_FePO_4_‐nanoplates	3.85 × 10^−11^	2.94 × 10^−11^

Figure 6c shows the EIS curves of Li_(4C‐40%)_FePO_4_ with different morphologies. Upon equivalent circuit fitting, the electrolyte resistance R1 of Li_(4C‐40%)_FePO_4_‐nanoparticles is 66.08 Ω and the charge transfer resistance R2 is 25.87 Ω; for Li_(4C‐40%)_FePO_4_‐nanorods, R1 is 42.75 Ω, and R2 is 10.83; for Li_(4C‐40%)_FePO_4_‐nanoplates, R1 is 11.35 Ω, and R2 is 12.76. These results indicate that the prelithiation process have reduced the energy barrier of Li^+^ de‐embedding for all materials with different morphologies. The variation of discharge specific capacity of FePO_4_ and Li_(4C‐40%)_FePO_4_ with different morphologies at 1 C in LiCl solution is shown in Figure 6d. The prelithiated electrode materials Li_(4C‐40%)_FePO_4_ with different morphologies all have higher discharge specific capacities than FePO_4_. The first discharge specific capacities were 64.3 mAh g^−1^ for FePO_4_‐nanoparticles and 78.8 mAh g^−1^ for Li_(4C‐40%)_ FePO_4_‐nanoparticles; the first discharge specific capacities were 68.4 mAh g^−1^ for FePO_4_‐nanorods and 80.9 mAh g^−1^ for Li_(4C‐40%)_FePO_4_‐nanorods. FePO_4_‐nanoplates had a specific capacity of 98.7 mAh g^−1^ for the first discharge, and Li_(4C‐40%)_ FePO_4_‐nanoplates had a specific capacity of 126.0 mAh g^−1^ for the first discharge. Compared to FePO_4_, Li_(4C‐40%)_FePO_4_ has an improved discharge specific capacitance, with the highest capacity in the Li_(4C‐40%)_FePO_4_‐nanoplates, which is attributed to the fact that the nanoplate structure usually has a larger surface area and a shorter electron conduction path, which is conducive to the improvement of the material's electron conduction properties. The rate capability of Li_(4C‐40%)_FePO_4_ with different morphologies at charge and discharge rates from 0.5 C to 4 C is shown in Figure S11 (Supporting Information). Li_(4C‐40%)_FePO_4_ consistently exhibits higher discharge specific capacity compared to FePO_4_ across various charging rates. The benefits of the prelithiated structure become more evident as the charge/discharge rate increases.

Figure 6e shows the potential and capacity curves of Li_(4C‐40%)_FePO_4_//AC systems with different morphologies with different morphologies in simulated brine. The unit lithium extraction amount and energy consumption of Li_(4C‐40%)_FePO_4_ with different morphologies are significantly different. The lithium extraction amount of one cycle of Li_(4C‐40%)_FePO_4_‐nanoparticles was 2.40 mmol g^−1^, and the lithium extraction energy consumption was 0.96 Wh mol^−1^. The lithium extraction amount of one cycle of Li_(4C‐40%)_FePO_4_‐nanorods was 3.76 mmol g^−1^ (Figure 6f), and the lithium extraction energy consumption was 0.80 Wh mol^−1^. The lithium extraction amount of Li_(4C‐40%)_FePO_4_‐nanoplates cycling one turn was 5.06 mmol g^−1^, and the lithium extraction energy consumption was 1.13 Wh mol^−1^. The lithium extraction purity of Li_(4C‐40%)_FePO_4_ with different morphologies reached a high level, which were (97.06%, 98.61%, and 98.85%), respectively (Figure 6g). It can be seen that Li_(4C‐40%)_FePO_4_‐nanoplates have the highest lithium extraction per unit, which is due to the fact that the lamellar structure has a shorter ion diffusion path in the [010] channel, which is favorable for the diffusion rate of Li^+^ inside the material. In addition, the larger surface area helps to increase the contact area between the material and the electrolyte, which promotes rapid ion transfer and improves the electrochemical reaction rate. The shape and low defect level of Li_(4C‐40%)_FePO_4_‐nanoparticles could increase the miscibility gap, which can suppress solid solution formation, demonstrating the weak lithium extraction performance among the three morphologies.

## 3 Conclusion

The FePO_4_ were subjected to prelithiation operation and the crystal structure, elemental distribution, morphology, and electrochemical properties of the materials before and after modification were compared. This work exploring the impact of particle sizes and morphologies of FePO_4_ on the prelithiation effect on lithium extraction performance. The study results indicated that the Li_(4C‐40%)_FePO_4_//AC system enhanced lithium extraction capacity and purity, as well as reduced energy consumption for lithium extraction compared to the FePO_4_//AC system. As the particle size increased, the energy consumption per unit of lithium extraction remained relatively stable, while the amount of lithium extracted per unit increased, maintaining a purity level above 97.93%. Specifically, the Li_(4C‐40%)_FePO_4_‐2500 demonstrated an energy consumption of 0.79 Wh mol^−1^ and a purity of 97.94% for lithium extraction at a unit lithium extraction of 5.93 mmol g^−1^ in simulated brine. Among the prelithiated iron phosphate materials with different morphologies, the lithium extraction purity was above 97.06%, in which the highest lithium extraction per unit of Li_(4C‐40%)_FePO_4_‐nanoplates was 5.06 mmol g^−1^, and the lowest lithium extraction energy consumption per unit of Li_(4C‐40%)_FePO_4_‐nanorods was 0.80 Wh mol^−1^. Furthermore, the diffusion energy barriers of Li^+^ and Na^+^ were calculated using DFT both before and after prelithiation, demonstrating good agreement with the experimental findings.

In summary, Li_(4C‐40%)_FePO_4_ demonstrates significant advantages in terms of selectivity and energy consumption. The extent of this prelithiation effect can be adjusted by modulating the morphology and particle size of iron phosphate nanostructures.

## 4 Experimental Section

### Chemicals and Materials

Lithium hydroxide (AR) and sodium dodecylbenzene sulfonate (AR) were obtained from Macklin. Ferrous sulfate (AR), nitrate tetrafluoroborate (96%), phosphoric acid (AR), and L‐ascorbic acid (reagent‐grade) were obtained from Aladdin.

### Synthesis of Materials—Synthesis of LiFePO_4_ with Different Particle Sizes

LiFePO_4_ was prepared using the precipitation method as follows: first, FeSO_4_·7H_2_O (1.32 m) was dissolved in H_3_PO_4_ and stirred at 25 °C for 30 min to ensure adequate mixing. Subsequently, H_2_O_2_ was added and the pH of the solution was adjusted to 2 using NaOH (1.25 m) solution. After that, the solution containing the precipitate was placed in an ambient environment at 90 °C with continuous stirring for 5 h. After completion of the stirring, the resulting precipitate was collected by filtration and washed several times with distilled water to remove impurities. Subsequently, the precipitate was dried in a vacuum oven at 110 °C for 10 h to remove residual water. It was transferred to a muffle furnace and calcined at 600 °C for 5 h. Next, in order to further optimize the properties of the material, the prepared precursor and glucose were mixed and transferred to a tube furnace and calcined at 750 °C for 5 h under N_2_ atmosphere with a temperature increase rate of 5 °C min^−1^. LiFePO_4_ synthesized via the co‐precipitation method was fractionated using high‐speed cryo‐centrifugation in ethanol solvent at rotational speeds of 300, 600, and 1200 rpm, resulting in the successful isolation of three materials with particle size distributions of 500, 1500, and 2500 nm.

### Synthesis of Materials—Synthesis of LiFePO_4_ with Different Morphology

LiFePO_4_ nanoplates, nanorods, and nanoparticles were prepared by a hydrothermal method, and the selected reactants included LiOH·H_2_O, FeSO_4_·7H_2_O, H_3_PO_4_, L‐ascorbic acid and sodium dodecylbenzene sulfonate (SDBS) with a stoichiometric ratio of 3:1:1:1:1:0.5:0.2. An aqueous solution of FeSO_4_·7H_2_O (0.015 m), H_3_PO_4_ (0.015 m), LiOH·H_2_O (0.045 m), and sodium dodecyl benzene sulfonate (0.003 m) was first mixed in 40 mL of distilled water with stirring, and then the mixture was transferred to a 50 mL PTFE‐lined stainless steel autoclave and heated for 24 h at 170 °C. After the hydrothermal reaction, the autoclave was cooled to room temperature, and the obtained precipitate was filtered, washed and dried at 60 °C for 12 h. For the synthesis of LiFePO_4_ nanorods, the other steps are the same with previous, except that the concentration of SDBS is reduced by 5 times. The synthesis process of LiFePO_4_ nanoparticles is also the same, but there is no SDBS in the reaction solution. In order to further enhance the conductivity of the materials, the prepared LiFePO_4_ was modified with carbon coating by heat treatment: Sucrose was first dissolved in distilled water, and finally prepared into a solution with a liquid‐solid ratio of 2:1(mL:g), and then mixed at room temperature using a 250 W, 40 kHz ultrasonic device for 20 min to form a uniform slurry. The slurry sample was dried at 90 °C for 12 h to remove excess water to obtain the final precursor. The precursor was first heated to 350 °C for 4 h (heating rate: 4 °C min^−1^) in a tube furnace under N_2_ atmosphere, and then heated to 650 °C for 9 h (heating rate: 2 °C min^−1^).^[^ ^77^, ^78^, ^79^ ^]^

### Synthesis of Materials—The Process of Prelithiation

The steps and schematics of material synthesis in the prelithiation process are shown in Figure S1 (Supporting Information). The chemical removal of lithium from carbon‐coated LiFePO_4_ consisted of the following steps: first, an oxidizing solution was prepared by dissolving 1.36 g of nitrate tetrafluoroborate in 80 mL of acetonitrile. Subsequently, 0.8 g of carbon‐coated LiFePO_4_ powder was immersed into the solution and stirred at room temperature (20–25 °C) for 24 h. After stirring, the powder was washed with acetonitrile several times, and finally dried in a vacuum oven for 12 h. Exploratory experiments, as illustrated in Figure S2 (Supporting Information), suggest that Li_x_FePO_4_ electrodes containing 40% Li content effectively reduce the Li^+^ insertion energy barrier, with a 4C insertion rate achieving optimal prelithiation. During the prelithiation process, the FePO_4_ working electrode was paired with a platinum mesh counter electrode, and 40% lithium was inserted at a 4 C insertion rate in 50 mL 1 m pure lithium chloride. After the prelithiation process, the electrode was rinsed with deionized water to remove the adsorbed Li^+^. Figure S3 (Supporting Information) shows the integral area of the constant current discharge curve of the empty host FePO_4_ embedded with 40% lithium and 100% lithium, respectively.

### Characterization

The crystal structure and lattice parameter of the materials were analyzed using X‐ray diffraction (XRD) (XRD‐6100, Japan, 2θ: 10°−80°, scanning rate: 5° min^−1^). Scanning electron microscopy (SEM) (FEl Apreo, USA) was utilized to further obtain information on the microscopic morphology and elemental distribution of the materials. The chemical valence states of the materials were analyzed by X‐ray photoelectron spectroscopy (XPS) (K‐Alpha, USA). The specific content of cations in the electrolyte after electrochemical lithium extraction was measured using inductively coupled plasma optical emission spectrometry (ICP‐OES) (ICAPQ, USA).

### Electrochemical Testing

N‐methyl pyrrolidone was used as the solvent. The active substance powder, acetylene black, and PVDF were mixed according to the mass ratio of 8:1:1, pulsed, and the slurry was uniformly coated on the carbon sheet (the area of the coated active substance was 1 cm^2^), and then dried at 40 °C for 24 h. The loading during the electrochemical tests was ≈4–8 mg cm^−2^. The changes in the quality of electrode sheets before and after coating were recorded for determining the mass of the active substance.

The electrochemical tests were performed using a three‐electrode system consisting of a lithium insertion electrode as the working electrode, an Ag/AgCl reference electrode and a platinum mesh counter electrode. The Li^+^ diffusion coefficient was determined by analyzing the cyclic voltammetry (CV) data at different scan rates in 1 m LiCl solution. The cycling stability of the materials was tested using the constant current charge/discharge method. The impedance characteristics of the electrode materials were measured using electrochemical impedance spectroscopy (EIS) under alternating current conditions.

### Electrochemical Lithium Extraction

Electrochemical lithium recovery takes place within an electrode pair system comprising an iron phosphate electrode and an AC electrode. The entire process is divided into two steps: charging and discharging. During the discharge process, Li^+^ from simulated brine solution is inserted into the iron phosphate electrode. In the charging process, the solution is changed to a 10 mm receiving solution, allowing the Li^+^ released from the lithium iron phosphate electrode to enter the receiving solution.

### Evaluation Methodology

According to the CV curve, the diffusion coefficient of the active material can be calculated by Equation (1): where *i*_p_ is the peak current (A) in the CV curve; *n* is the gain and loss of electrons in the electrode reaction process; A is the effective electrode area of the electrochemical reaction (cm^2^); *D*_0_ is the diffusion coefficient of the active reactive material (cm^2^/s); *v* is the scan rate (V/s); and *c*_0_ is the initial reactant concentration (mol/cm^3^).

(1) $$\;i_{p}=\;\left(2.69\times 10^{5}\right)n^{3/2}AD_{0}^{1/2}v^{1/2}c_{0}$$

The extraction amount of Li^+^, *Q_r_*, can be calculated according to Equation (2): where *m* is the mass of the active substance, *V*_r_ is the volume of the receiving solution (L), *C*_r_ is the final concentration of Li^+^ in the receiving solution (mol/L), and *C*_1_ is the initial concentration of Li^+^ in the receiving solution (mol/L).

(2) $$\;Q_{r}=\frac{\left(C_{r}-C_{1}\right)}{m}\;\times V_{r}$$

The separation coefficient “λ” can be calculated from the final concentration of Li^+^ in the receiving solution, *C*_Li_, versus the final concentration of Na^+^, *C*_Na_, by using Equation (3).

(3) $$\;\lambda_{\mathrm{Li}-\mathrm{Na}}={\left(\frac{C_{Li}}{C_{Na}}\right)}_{t}\;\times {\left(\frac{C_{Na}}{C_{Li}}\right)}_{0}$$

The purity of Li^+^ *P*_Li_ is calculated by Equation (4), which is the percentage (%) of the Li^+^ concentration *C*_r_ extracted from the receiving solution divided by the total cation concentration (∑*C*_i_) extracted from the receiving solution.

(4) $$P_{Li}\left(\%\right)\;=\frac{C_{r}}{\sum C_{i}}\;\times 100$$

According to Equation (5), the energy consumption can be calculated by integrating the charge‐discharge curve: where Δ*E* is the change of voltage (V), *q* is the charge (C).

(5) $$W=\frac{\oint \Delta E•dq}{\left(C_{r}-C_{1}\right)\times V_{r}}\;$$

### DFT Calculation

Based on DFT, the diffusion processes of Li^+^ and Na^+^ in FePO_4_ and Li_(4C‐40%)_FePO_4_ structures, respectively, were simulated using DS‐PAW software.^[^ ^80^ ^]^

The calculation details are as follows: The generalized gradient approximation (GGA) was carried out by using the projector‐augmented wave (PAW) pseudopotential and the exchange‐correlation functional parameterized by Perdew–Burke–Ernzerhof (PBE). Due to the localization of d orbital electrons of transition metal ions, the standard DFT‐GGA method lacked the accuracy of dealing with strongly correlated materials (such as transition metal oxides or rare earth compounds). Considering the strong in‐situ coulomb interaction in the localized 3D electrons of Fe, the GGA+U method was used to deal with Fe‐3d electrons with localized characteristics. The effective interaction parameter U_eff_ was set to 4.3 eV. The wave function was expanded according to the plane wave base, using a plane‐wave cutoff of 520.0 eV. Brillouin zone integration was carried out by using k‐point of 5 × 2 × 2 Monkhorst–Pack type. All structures were sufficiently relaxed until the force on each atom is less than 0.03 eV Å^−1^ and the energy converges to less than 1 × 10^−5 ^eV per atom.

## Conflict of Interest

The authors declare no conflict of interest.

## Supporting information



Supporting Information

## Data Availability

The data that support the findings of this study are available from the corresponding author upon reasonable request.
