# Supplementary material for: Enhanced Lithium Extraction from Brines: Prelithiation Effect of FePO4 with Size and Morphology Control
Source: Adv Sci (Weinh). 2024 Sep 17;11(41):2405176. doi: 10.1002/advs.202405176 (PMC11538655; doi:10.1002/advs.202405176)
Supplement: Supplementary file 1 — Supporting Information [file ADVS-11-2405176-s001.docx]

**Enhanced Lithium Extraction from Brines: Pre-lithiation Effect of FePO_4_ with Size and Morphology Control**

Xiaoyu Zhao ^a,b,^*, Shuo Yang ^b^, Xiuli Song ^b^, Yushuang Wang ^a^, Hui Zhang ^a^, Muhan Li ^a^, Yanfei Wang ^b^

a, *State Key Laboratory of Biobased Fiber Manufacturing Technology, Tianjin University of Science and Technology, China*

b, *Tianjin Key Laboratory of Brine Chemical Engineering and Resource Eco-utilization, College of Chemical Engineering and Materials Science, Tianjin University of Science and Technology, China*

* Corresponding author: Xiaoyu Zhao (xyz@tust.edu.cn)

*
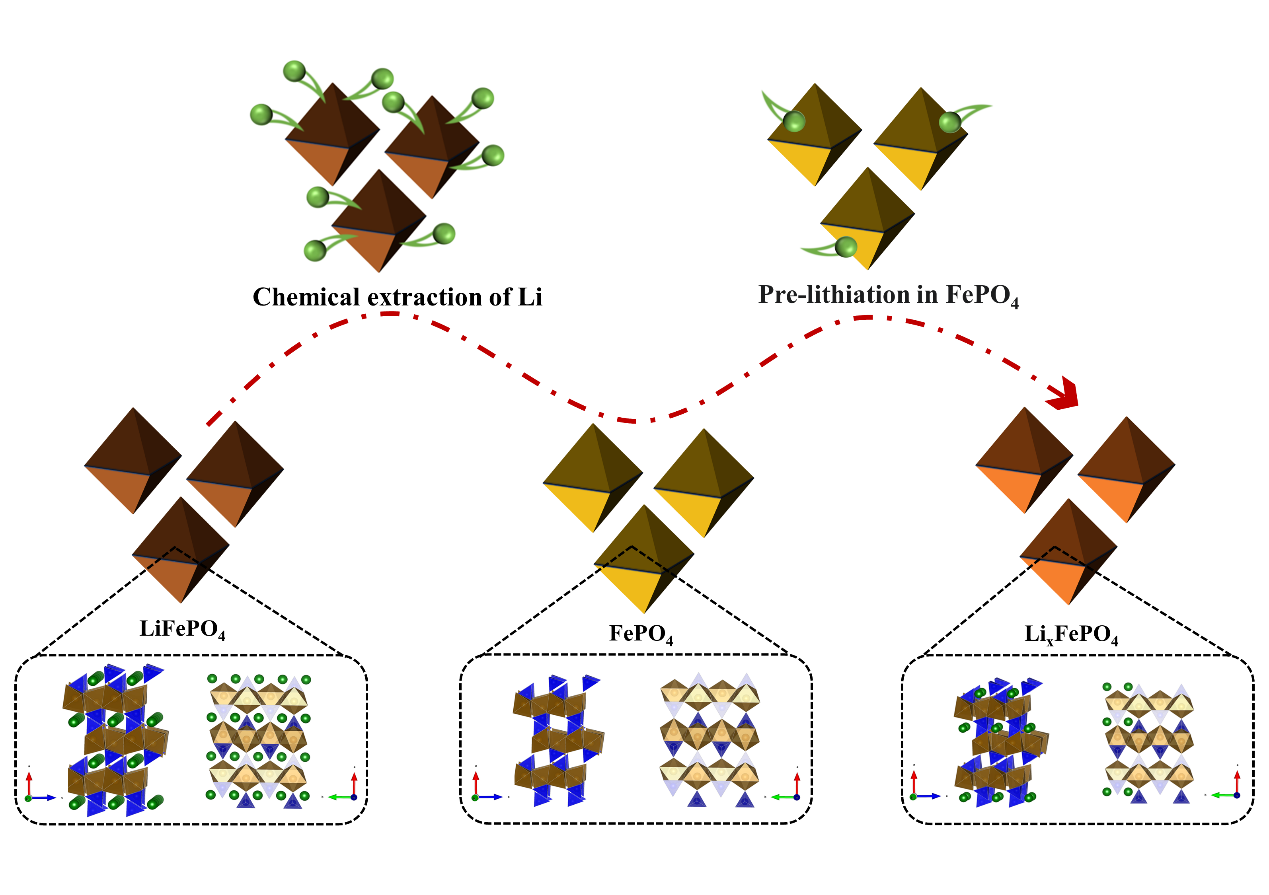
*

Fig. S1 Schematic diagram of material synthesis.

Fig. S2 Discharge capacity of Li_x_FePO_4_ at different Pre-lithiation current and Pre-lithiation content.

Fig. S3 Comparison of integral area of discharge curve of FePO_4_ embedded with 40% lithium and embedded with 100% lithium.

Fig. S4 XPS full spectrum of FePO_4_ and Li_(4C-40%)_FePO_4_.


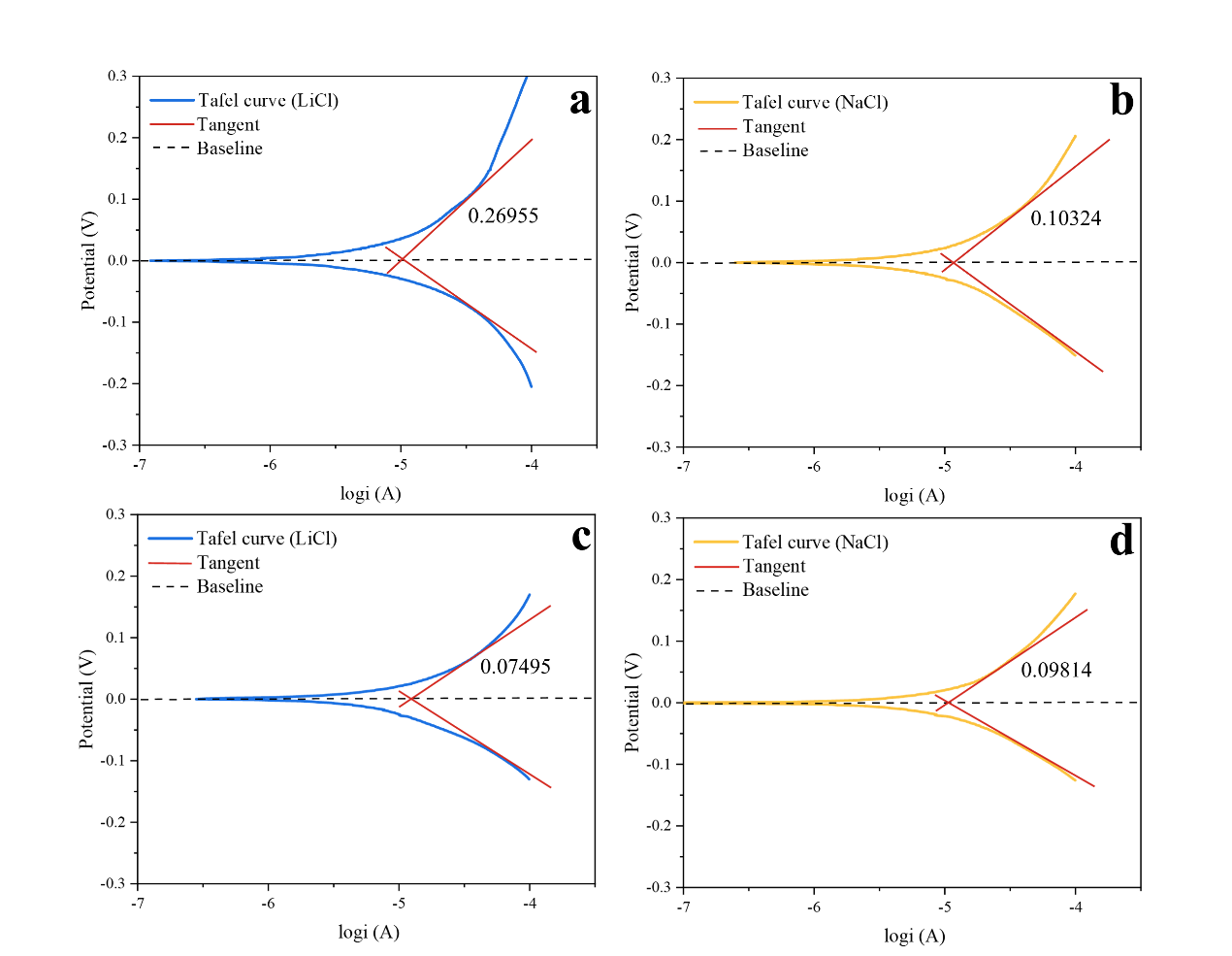


Fig. S5 Tafel curves of FePO_4_ (a) in 1M LiCl and (b) in 1M NaCl. Tafel curves of Li_(4C-40%)_FePO_4_ (c) in 1M LiCl and (d) in 1M NaCl.


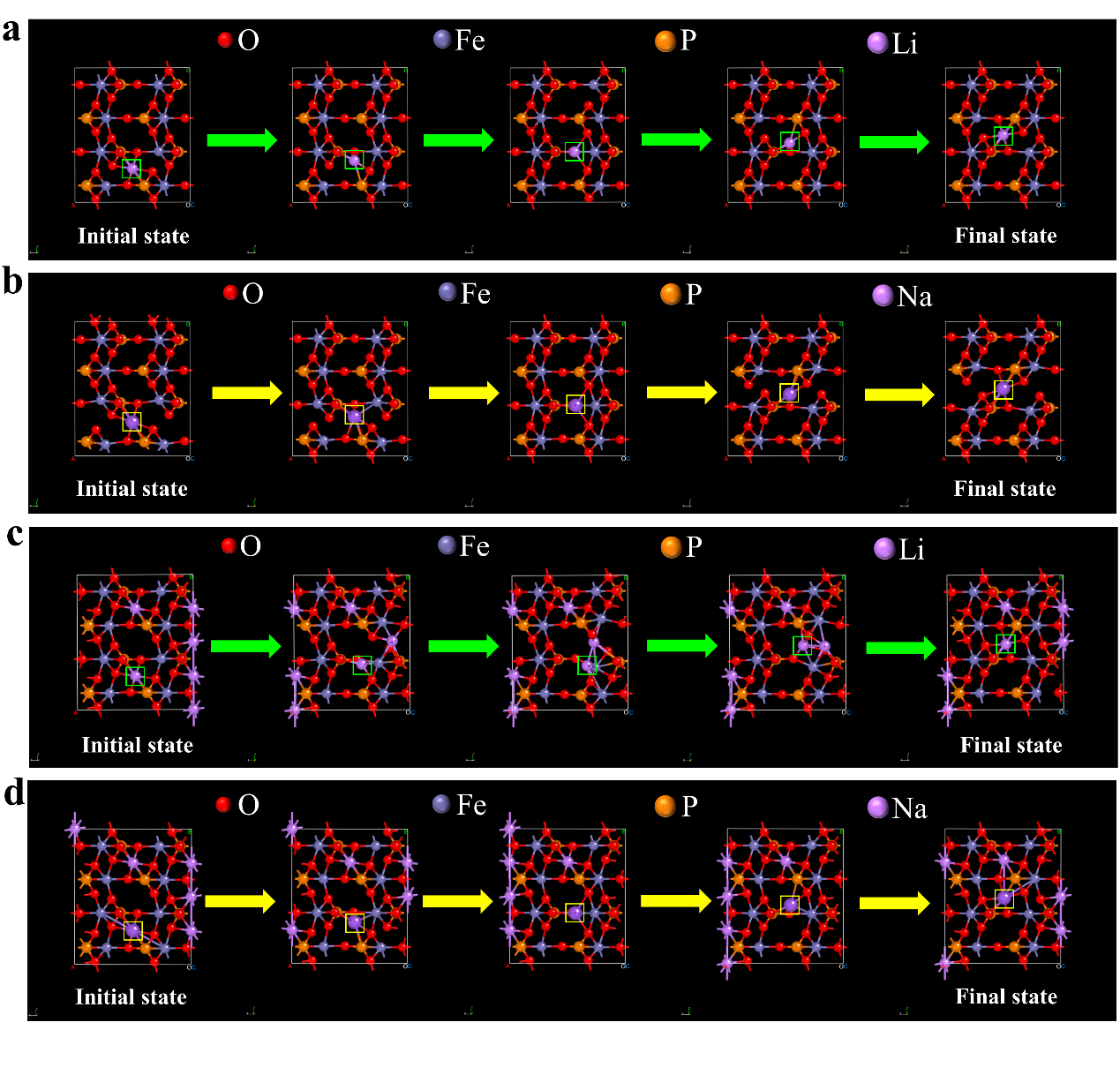


Fig. S6 (a) Diffusion path of Li^+^ in FePO_4_ structure. (b) Diffusion path of Na^+^ in FePO_4_ structure. (c) Diffusion path of Li^+^ in Li_(4C-40%)_FePO_4_ structure. (d) Diffusion path of Na^+^ in Li_(4C-40%)_FePO_4_ structure.


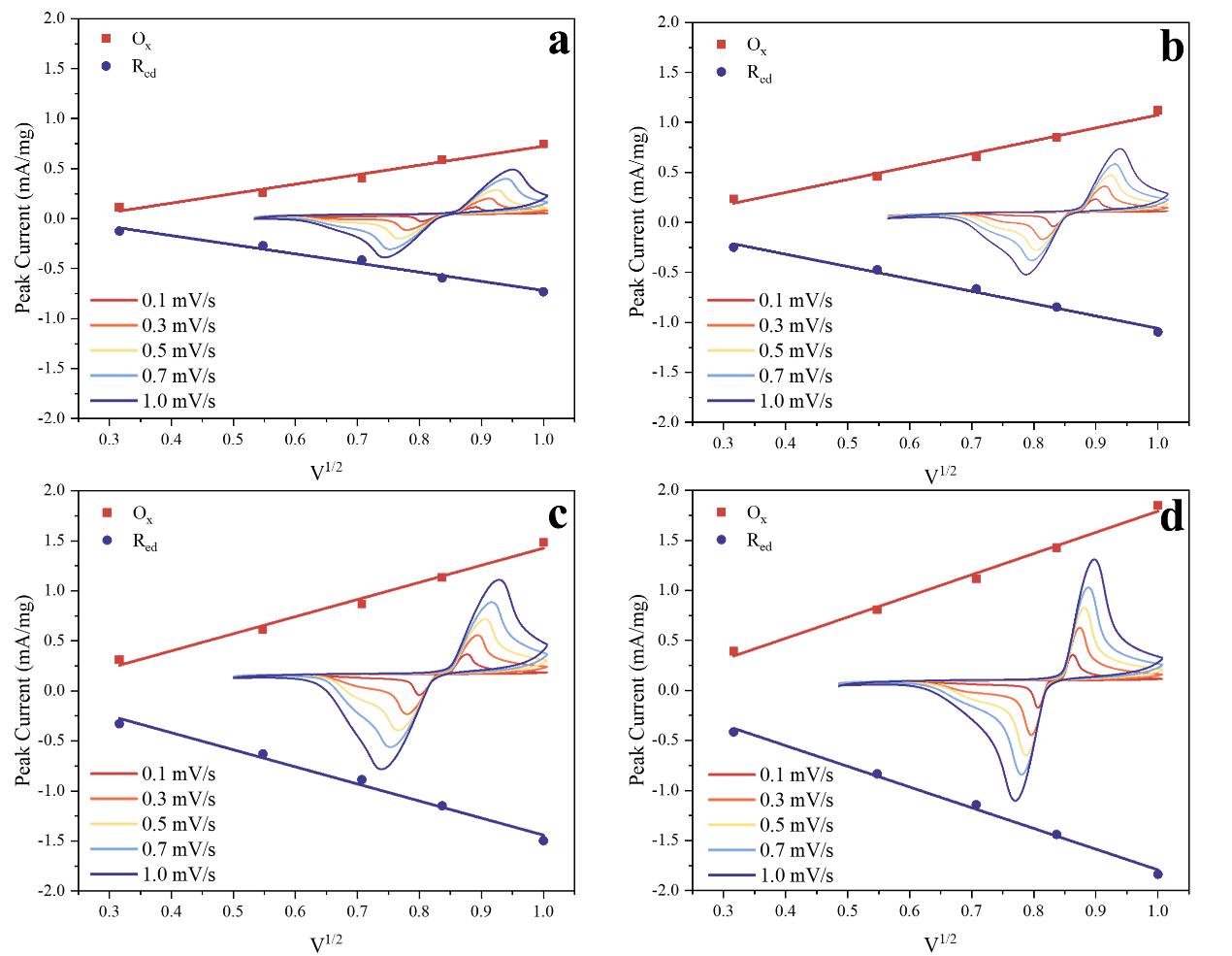


Fig. S7 CV curves at different scan rates in 1 M LiCl electrolyte and fitting plots of Ip to V^1/2^. (a) FePO_4_ (b) Li_(4C-40%)_FePO_4_-500 (c) Li_(4C-40%)_FePO_4_-1500 (d) Li_(4C-40%)_FePO_4_-2500.

Fig. S8 Rate capability at incremental discharge rates from 0.5 C to 4 C of FePO_4_ and Li_(4C-40%)_FePO_4_(500 nm, 1500 nm, 2500 nm).


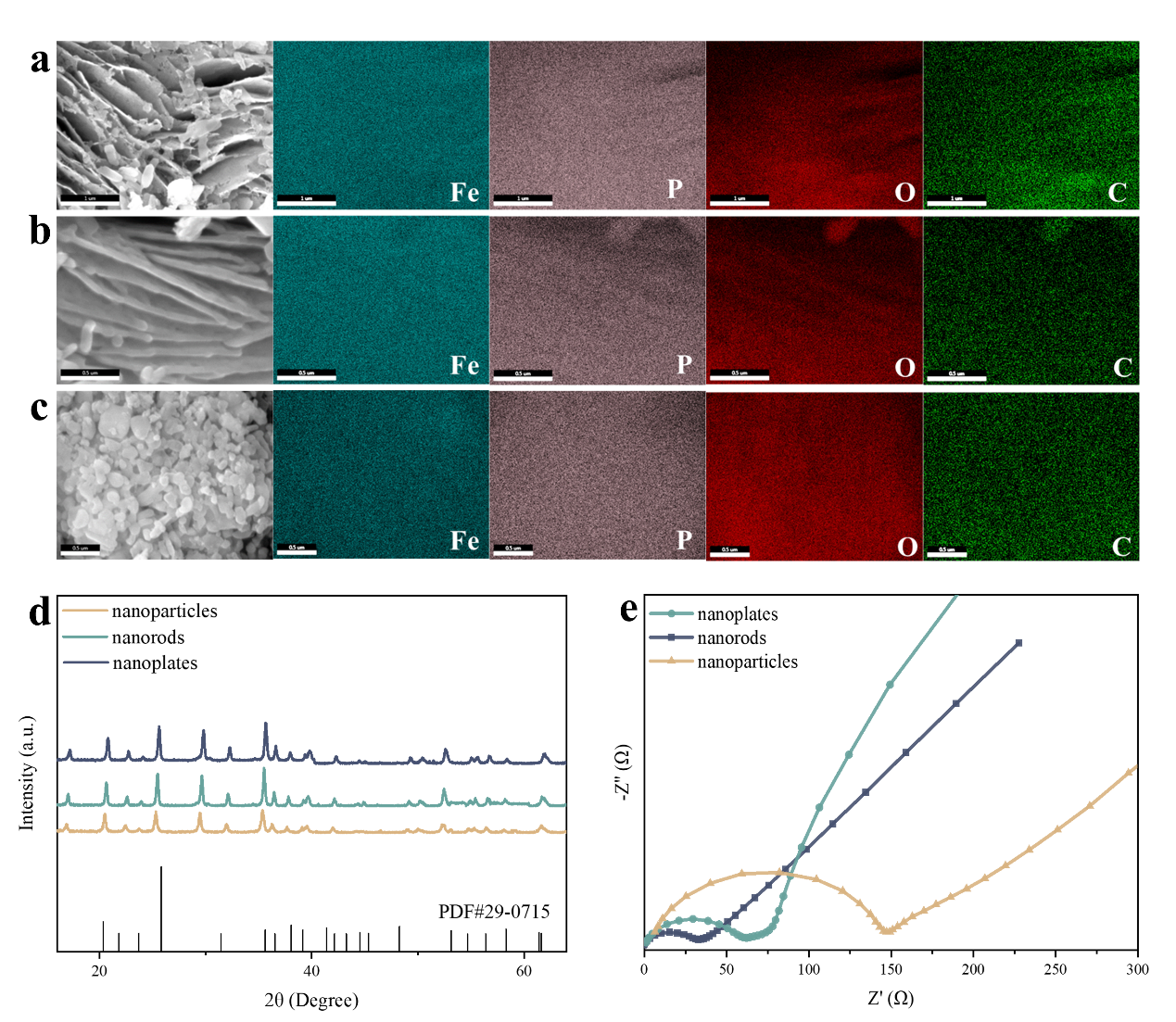


Fig. S9 EDS mapping of (a) LiFePO_4_-nanoplates, (b) LiFePO_4_-nanorods and (c) LiFePO_4_-nanoparticles. (d) XRD patterns of LiFePO_4_(nanoparticles, nanorods, nanoplates). (e) EIS curves of LiFePO_4_(nanoparticles, nanorods, nanoplates) electrodes.


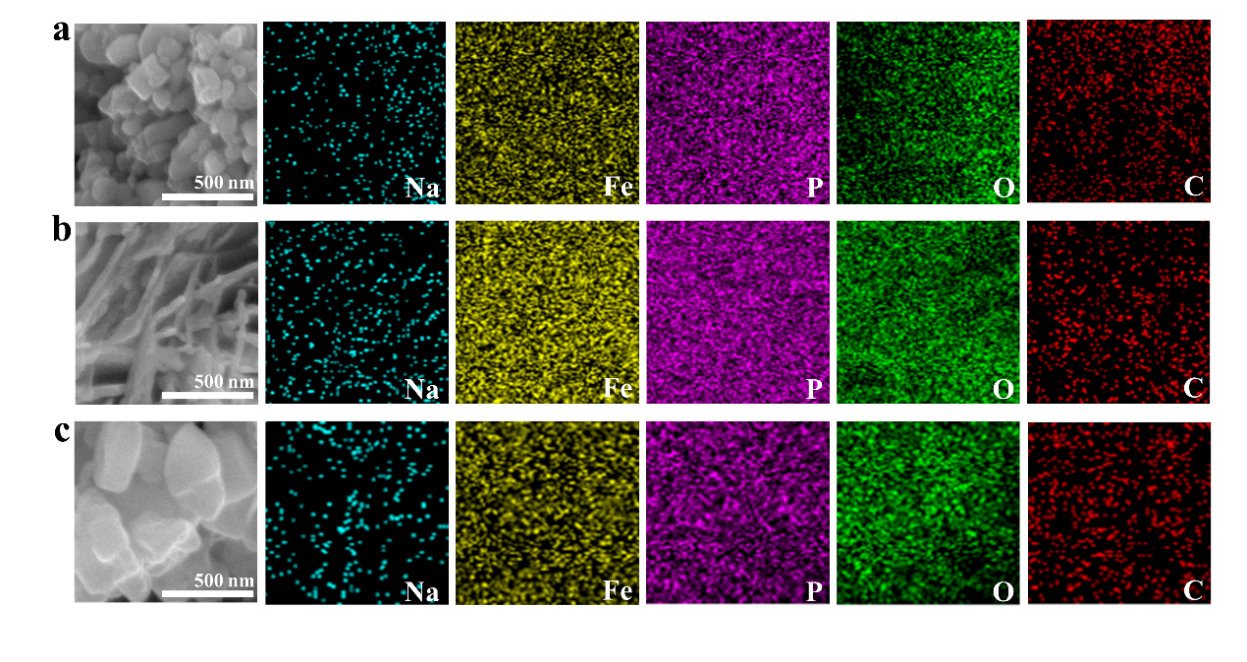


Fig. S10 EDS mapping of (a) Li_x_Na_y_FePO_4_-nanoparticles, (b) Li_x_Na_y_FePO_4_-nanorods and (c) Li_x_Na_y_FePO_4_-nanoplates.


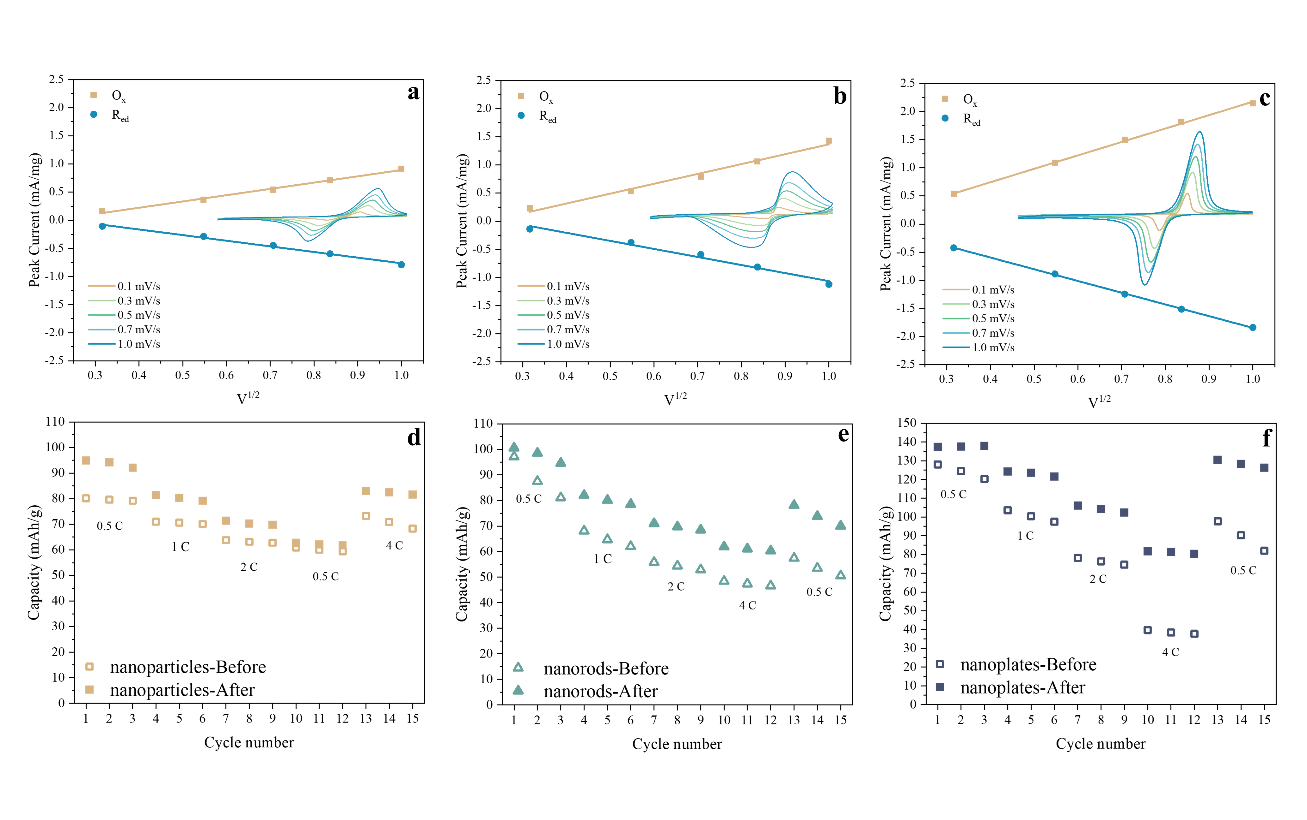


Fig. S11 CV curves at different scan rates in 1 M LiCl electrolyte and fitting plots of Ip to V^1/2^ (a) Li_(4C-40%)_FePO_4_-nanoparticles, (b) Li_(4C-40%)_FePO_4_-nanorods and (c) Li_(4C-40%)_FePO_4_-nanoplates. Rate capability at incremental discharge rates from 0.5 C to 4 C of (d) Li_(4C-40%)_FePO_4_-nanoparticles, (e) Li_(4C-40%)_FePO_4_-nanorods and (f) Li_(4C-40%)_FePO_4_-nanoplates.
